# The Differential Expression of CD47 may be Related to the Pathogenesis From Myelodysplastic Syndromes to Acute Myeloid Leukemia

**DOI:** 10.3389/fonc.2022.872999

**Published:** 2022-03-31

**Authors:** Xiao Yan, Binbin Lai, Xuyan Zhou, Shujun Yang, Qunfang Ge, Miao Zhou, Cong Shi, Zhijuan Xu, Guifang Ouyang

**Affiliations:** ^1^ Haematology Department of Ningbo First Hospital, Ningbo Clinical Research Center for Hematologic Malignancies, Ningbo, China; ^2^ Haematology Department, The Affiliated Hospital of Medical School of Ningbo University, Ningbo, China; ^3^ Medical Research Center of Ningbo First Hospital, Ningbo, China; ^4^ Stem Cell Transplantation Laboratory of Ningbo First Hospital, Institute of Hematology of Ningbo First Hospital, Ningbo, China

**Keywords:** mds, AML, cd47, DNA methylation, expression

## Abstract

Myelodysplastic syndrome (MDS) can lead to the development of peripheral blood cytopenia and abnormal cell morphology. MDS has the potential to evolve into AML and can lead to reduced survival. CD47, a member of the immunoglobulin family, is one molecule that is overexpressed in a variety of cancer cells and is associated with clinical features and poor prognosis in a variety of malignancies. In this study, we analyzed the expression and function of CD47 in MDS and AML, and further analyzed its role in other tumors. Our analysis revealed significantly low CD47 expression in MDS and significantly high expression in AML. Further analysis of the function or pathway of CD47 from different perspectives identified a relationship to the immune response, cell growth, and other related functions or pathways. The relationship between CD47 and other tumors was analyzed from four aspects: DNA methyltransferase, TMB, MSI, and tumor cell stemness. Changes in gene expression levels have a known association with aberrant DNA methylation, and this methylation is the main mechanism of tumor suppressor gene silencing and clonal variation during the evolution of MDS to AML. Taken together, our findings support the hypothesis that the differential expression of CD47 might be related to the transformation of MDS to AML.

## Introduction

Myelodysplastic syndromes (MDS) belong to clonal hematopoietic stem cell disorders that present with ineffective hematopoiesis, including peripheral cytopenia and abnormal cell morphologies (1). The World Health Organization (WHO) subdivides MDS patients’ risk levels from low to high according to the molecular and morphological characteristics, the number of primitive cells in the bone marrow, and the degree of peripheral cytopenia (2). Age differences are evident in the prevalence of MDS, as MDS is uncommon in children or adolescents. By contrast, a progressive increase occurs in its incidence in people between the ages of 40 and 80 years (3). Another hematopoietic disease involving myeloid or multipotent progenitor cells is acute myeloid leukemia (AML), a malignant clonal disease with molecular heterogeneity. The various clonal disorders associated with AML are caused by a failure in differentiation and an uncontrolled proliferation of hematopoietic progenitor cells, leading to the accumulation of many different cytogenetic disorders (4).

Both MDS and AML are clinically genetically heterogeneous myeloid stem cell disorders, and patients with MDS are at a high risk of progression to AML (5). Patients whose MDS progresses to AML have a lower response to standard therapy compared to patients with new-onset AML (6). In recent years, many new drugs have emerged to treat MDS, and allogeneic HSCT provides possibility to cure MDS (7). However, the stringent conditions for transplantation and the conversion to AML experienced by some patients have led to poor overall survival (OS) in MDS (8). This has prompted a search for the genetic basis of this conversion. One potential molecule that may be involved is Cluster of differentiation 47 (CD47).

CD47 is a cell surface glycoprotein molecule belonging to the immunoglobulin superfamily. It is a widely expressed transmembrane protein in human cells and is overexpressed on the surface of many cancer cells. Its binding to signal-regulated protein α (SIRPα) signals cancer cells to escape from macrophage-mediated phagocytosis (9), thereby promoting tumor progression. CD47 expression has also been associated with the clinical features and prognosis of a variety of malignancies; for example, its expression is associated with poor prognosis and the pathological features of colorectal cancer (10) and it affects the progression-free survival (PFS) of patients with non-small cell lung cancer (11). CD47 also appears to have an important role in several hematological malignancies, such as acute lymphoblastic leukemia (ALL) and AML (12). Clinical studies of CD47 antibodies or targeted drugs for MDS or AML have been carried out, but most have been based on blocking CD47 expression to restore the phagocytosis of foreign cells by macrophages.

In the present study, we examined the role of CD47 from the perspective of the transformation of MDS to AML. In recent years, the development of genetic testing technology has greatly assisted the study of the genetic landscapes of diseases and their relationship to the pathogenesis and progression of diseases such as MDS (13). Comprehensive genomic analysis of MDS and AML has enabled the detection and differentiation of drivers and subclonal mutations, while informing risk prediction and defining targeted therapies (14). Here, we used high-throughput microarray technology and comprehensive bioinformatics analysis to explore the potential relationships between CD47 expression and functional pathways in MDS and AML.

## Data and Methods

### Data Sources

Data microarrays related to MDS and AML were obtained from the Gene Expression Omnibus (GEO) database. The gse30029 dataset included 90 AML samples and 31 normal control samples, the gse24395 dataset included 12 AML samples and 5 normal control samples, the gse10695 dataset included 20 MDS samples and 10 normal control samples, and the gse30195 dataset included 15 MDS samples and 4 normal control samples. Bone marrow RNA-seq data for AML were obtained from the Target database for a total of 240 cases. RNA-seq data of tumors were obtained from The Cancer Genome Atlas (TCGA) database and paraneoplastic data from the GTEx dataset. Tumor cell sequencing data were obtained from CCLE for 27 tissues.

### Gene Expression Analysis

The distribution of CD47 expression in the two groups of samples was analyzed using the Wilcoxon rank sum test, and violin plots were drawn with the R package ggplot2. Spearman’s correlation analysis was used to describe the correlation between quantification and variables without normal distribution, and the correlation maps of genes were presented using the R package pheatmap. A value of p<0.05 was considered statistically significant, with an absolute value of the correlation coefficient closer to 1, indicating a stronger correlation.

### Analysis of Differences

Microarrays for MDS or AML were obtained from the GEO database, and samples were categorized into disease or control groups based on clinical information. Differential expression of mRNA was analyzed using the R package limma package with P<0.05, and |log2FC|>1 was defined as the screening threshold for differentially expressed genes (DEG). The results of the differential analysis for each data set were presented using volcano plots. The overlap of the two DEG sets was then used in subsequent analysis.

### Protein-Protein Interaction Network

We analyzed the functional pathways common to MDS and AML by PPI analysis of the overlapping genes. The STRING database (https://string-db.org/) was used to obtain the network relationship map of the overlapping genes, and the key functional gene modules were obtained by the MCODE plug-in in Cytoscape software. We used GeneMANIA (https://genemania.org/) to construct a PPI network centered on CD47, which included association data for protein and genetic interactions, pathways, co-expression, co-localization, and protein structural domain similarity.

### Functional and Pathway Enrichment Analysis

Metascape (https://metascape.org/gp/index.html#/main/step1), a website for analyzing gene or protein lists, was used to analyze the functional clustering of gene sets. The R package ClusterProfiler package was used to analyze gene sets for gene ontology (GO) and the Kyoto Encyclopedia of Genes (KEGG), and P<0.05 was considered significant. Gene set enrichment analysis (GSEA) was performed to investigate the biological signaling pathways between high and low CD47 expression. |NES|>1,NOM p-val<0.05,FDR q-val<0.25 were the pathway screening thresholds.

## Results

### Expression Distribution of CD47

**Figure 1:**
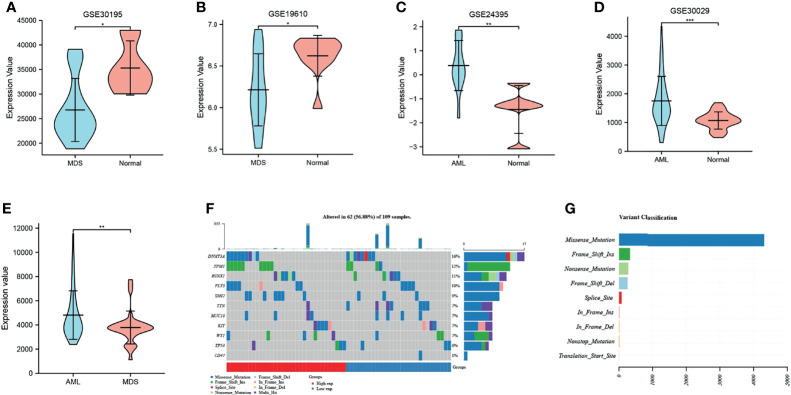
The expression and mutation of CD47. **(A, B)** CD47 expression analysis in MDS and normal control samples with datasets GSE30196 and GSE19610; **(C, D)** CD47 expression in AML and normal control samples using the GSE24395 and GSE30029 data sets. *P < 0.05, **P < 0.01, ***P < 0.001. **(E)** expression level of CD47 in AML and MDS. **(F)** Oncoplot shows the somatic landscape of acute myeloid leukemia. **(G)** Variant classification of CD47 mutations.

Further analysis of the CD47 expression levels in MDS and AML using GEO microarrays revealed expression in MDS tissues (**Figures 1A, B**) and high expression in AML tissues (**Figures 1C, D**) compared to control tissues. Integrate the data of these four chips and eliminate outlier samples to observe the expression of CD47. It was found that the expression level of CD47 in AML was significantly higher than that in MDS (**Figure 1E**). The waterfall diagram shows the mutations of 10 genes in AML. Compared with other genes, the mutation frequency of CD47 is not high, only 1% (**Figure 1F**). However, we found that mutations in CD47 involved only one variant classification of missense mutations (**Figure 1G**). This mutation usually causes protein abnormalities.

### Functional Analysis of CD47-Related Genes

**Figure 2:**
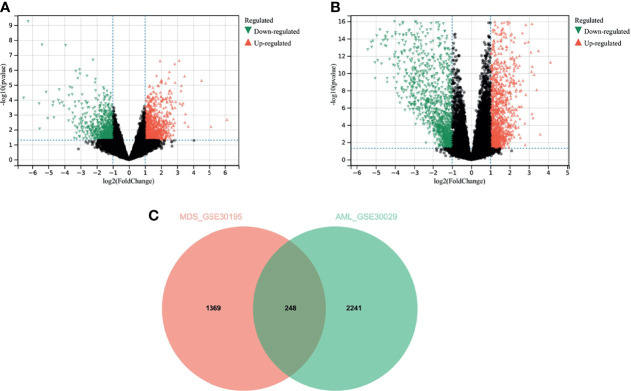
Results of analysis of variance. **(A)** analysis of variance for the MDS dataset GSE30195; **(B)** analysis of variance for the AML dataset GSE30029; **(C)** Venn diagram for the overlapping genes of the two sets of DEGs.

A PPI network of 21 genes centered on CD47 was constructed using GeneMANIA (**Figure 2A**). GO functional enrichment and KEGG pathway analysis were performed for these 21 genes. Significantly enriched GO terms included leukocyte migration, cell adhesion and activation, and integrin-mediated signaling pathways (**Figure 2B**), while significantly enriched KEGG pathways included EMC receptor interactions, adherent spots, human papillomavirus infection, and PI3K-Akt signaling pathway (**Figure 2C**).

### Identification and Analysis of DEGs

**Figure 3:**
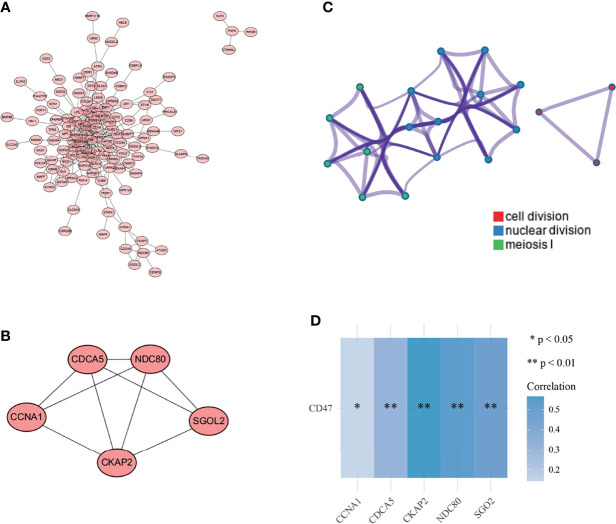
Selection of key gene modules. **(A)** PPI network of overlapping genes; **(B)** gene modules with the highest tightness were analyzed by MCODE plug-in **(C)**; functional clustering network map of overlapping genes; **(D)** correlation analysis of CD47 with 5 key genes in the target database (sgol2 is also called sgO2). *P < 0.05, **P < 0.01.

We used differential analysis to discern the common functions and pathways in MDS and AML disease progression. Overall, 693 downregulated genes, and 924 upregulated genes were identified in GSE30195 (**Figure 3A**) and 1327 downregulated genes and 1162 upregulated genes in GSE30029 (**Figure 3B**). The Venn diagram yielded 248 overlapping genes that were differentially expressed in both MDS and AML (**Figure 3C**).

### Function of Key Gene Modules

**Figure 4:**
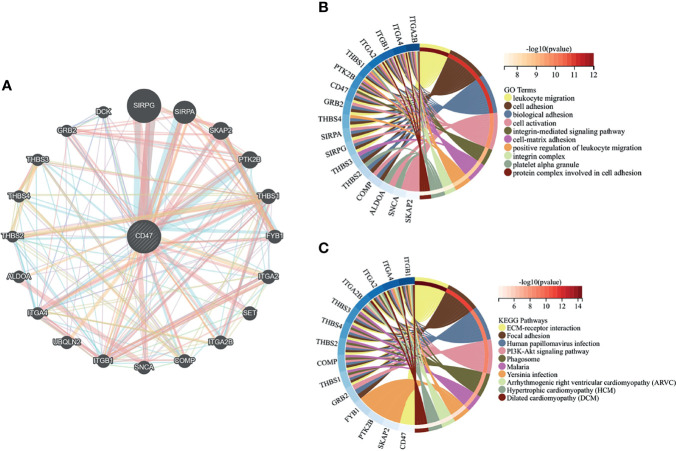
Gene interaction network and enrichment analysis. **(A)** Construction of a PPI network with 21 genes centered on CD47; **(B)** Top 10 GO functional terms; **(C)** Top 10 KEGG pathways.

The functional relationships of 248 overlapping genes were presented as PPI network diagrams (**Figure 4A**). Subsequently, four modules were obtained with the MCODE plug-in, and module 1 was selected as the key gene module (**Figure 4B**). The module was scored as 4.5 and contained 5 nodes and 9 edges, each node representing a gene. Enrichment analysis of these 5 genes was performed using Metascape, with each color indicating a functional cluster (**Figure 4C**). The significantly enriched functional terms include cytokinesis, nuclear division, and meiosis I phase. The relationship between CD47 and these five genes was analyzed using the Spearman correlation, and CD47 showed a varying degree of correlation with each gene; the greatest correlation was with CKAP2 (**Figure 3D**).

### Functional Pathways of CD47 in MDS and AML

**Figure 5:**
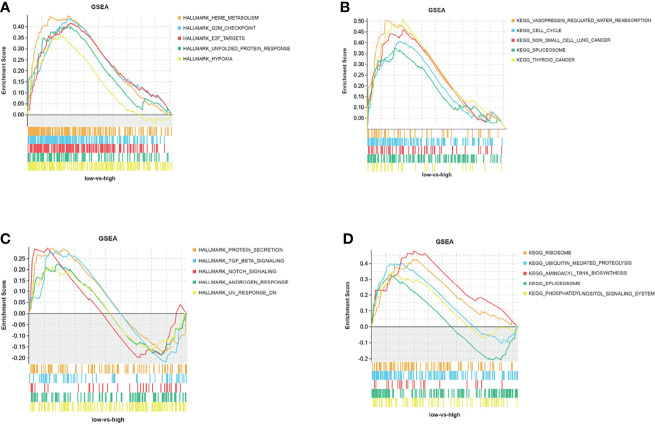
GSEA analysis of CD47 Hallmark and KEGG pathways. **(A)** Hallmark and **(B)** KEGG pathways of CD47 in MDS tissues from the GSE30195 dataset, and **(C)** hallmark and **(D)** KEGG pathways of CD47 in AML tissues from the GSE30029 dataset.

The GSEA results showed that the CD47 high expression group in MDS tissues was mainly enriched in Hallmark pathways, such as heme metabolism, G2M checkpoint, and E2F target (**Figure 5A**), and in KEGG pathways, such as pressin-regulated water reabsorption, cell cycle, and spliceosome (**Figure 5B**). By contrast, CD47 in AML tissues was mainly enriched in Hallmark pathways, such as protein secretion, TGF-β signaling, and NOTCH signaling (**Figure 5C**), and KEGG pathways, such as ribosomes, ubiquitin-mediated protein hydrolysis, and amyl-TRNA biosynthesis (**Figure 5D**).

## Discussion

In recent years, immunotherapy has shown a therapeutic effect against a variety of malignancies. One of the key cell types in the innate immune response is the macrophage, and cells expressing CD47 have the ability to evade the clearance by macrophages and other phagocytes. Consequently, CD47 is considered to be a main macrophage checkpoint (15). Given this relationship between CD47 and macrophages, many studies are now analyzing the roles of CD47 in tumor immunotherapy.

CD47 has shown potent anticancer potentials in a variety of hematologic malignancies, including MDS and AML, and a number of CD47-related antibodies or target drugs have emerged. For example, the humanized anti-CD47 monoclonal antibody CC-90002 enables tumor cells killed by macrophages by blocking the CD47/SIRPα interaction (16). Similarly, a SIRPα-αCD123 antibody, reported by Siret Tahk et al, intervenes against tumors by blocking local CD47 and binding to a single molecule on specific leukemic stem cells (17). These studies have revealed the mechanisms of actions of CD47 in MDS or AML from a horizontal perspective, whereas the present study explored the effect and association of CD47 expression on the transformation of MDS to AML from a longitudinal perspective.

MDS is a set of diseases associated with ineffective hematopoiesis, which is characterized by increased apoptosis of early and mature hematopoietic cells (18). In clinical practice, MDS is considered to transition to AML when the number of bone marrow blasts exceeds20% (19). In MDS, DNA methylation or DNA repair, chromatin modification, RNA splicing, signal transcription, and mucin regulation are the main mutational targets, and these mutational modalities share a common clonal origin with AML (20). This is because mutations in MDS and AML allow a well-organized expansion of the initiating clone, while the functional interactions that exist between mutations also determine disease progression.

Many studies now consider mutational events involving active signaling, myeloid transcription, or tumor suppressors as necessary for the progression of MDS to AML (21). A clear example is the difference in the mutation frequency of certain genes in MDS and AML. For example, some genes, such as receptor tyrosine kinases (FLT3 and KIT) and RAS pathway genes, have a higher proportion of mutations in AML, whereas mutations in splicing factors (SF) and epigenetic regulators, among others, are more prevalent in MDS (20). Another major cause of conversion of MDS to AML is DNA methylation (22), an epigenetic modification that regulates gene expression and is a key event in tumorigenesis (23). DNA methylation involves the covalent bonding of a methyl group at the cytosine 5’ carbon position of the genomic CpG dinucleotide in the presence of DNA methyltransferase. Many malignancies, including AML and MDS, typically exhibit aberrant DNA methylation and altered histone modifications that result in altered gene expression (24). For example, a study by Wen Jing Ding et al. demonstrated that upregulation of the expression of RAP1GTPase activating protein 1 (Rap1GAP), a gene involved in hematopoietic regulation, was associated with a lower methylation status of the promoter region of this gene in MDS patients (25).

CD47 has been reported to show high expression in AML tissues, and higher CD47 mRNA expression is an independent factor for poor prognosis in AML patients (26). One study showed that CD47 expression in MDS gradually increased with the evolution of risk scores in the International Prognostic Scoring System (IPSS-R), suggesting that CD47 expression levels may contribute to the progression from MDS to AML (27). In this study, CD47 was found to be significantly expressed in AML but poorly expressed in MDS. Meanwhile, the expression of CD47 correlated with DNA methyltransferase in AML. Therefore, we hypothesized that this differential expression of CD47 was associated with an aberrant DNA methylation status.

In general, DNA hypermethylation induces transcriptional repression of oncogenes, while hypomethylation induces activation of oncogenes (28). However, increased expression of promoter methylation-regulated genes occasionally occurs (29). Demethylation drugs have been used clinically to treat patients with MDS, and the methylation of certain oncogenes is confirmed to lead to the development or progression of MDS (30). Aberrant DNA methylation is also the main mechanism of tumor suppressor gene silencing and clonal variation during the evolution of MDS to AML (31). Thus, aberrant DNA methylation may induce the transformation of MDS to AML by altering the expression of CD47, although this speculation still needs confirmation by further studies.

In addition, the functional pathways of key genes were analyzed. The results showed that the genes associated with AML and MDS were significantly enriched in cell cycle related signal pathways including cell division. Coincidentally, GSEA results showed that CD47 was also related to cell growth, cell cycle and other related signal pathways in AML and MDS. The activation of CD47 may induce the growth of tumor cells and accelerate the proliferation and transformation (32). It is reported that the increase of cell proliferation is conducive to cell mutation and leukemia transformation (33). Misreplication during cell division can lead to the increase of mutation load and the loss and accumulation of methylation, which will also coordinate the regulation of cell cycle during tissue formation (34, 35). These evidences suggest that abnormal cell cycle is closely related to the cell mutation and DNA methylation. However, there is no final conclusion whether the expression of CD47 promotes the transformation of acute myeloid leukemia by affecting cell cycle related pathways. This study puts forward this assumption here, but the specific mechanism still needs to be verified by in further experiments.

In this study, we analyzed the expression of CD47 in MDS and AML and conducted a functional pathway analysis of CD47. We found that CD47 was differentially expressed in MDS and AML, and the difference in CD47 expression may reflect an abnormal DNA methylation status, which may be associated with the conversion of MDS to AML.

## Data Availability Statement

The original contributions presented in the study are included in the article/supplementary material, further inquiries can be directed to the corresponding author.

## Author Contributions

All authors contributed to the article and approved the submitted version.

## Funding

Zhejiang Provincial Health Science and Technology Program 2022KY316; 2021KY997; Zhejiang Provincial Natural Science Foundation LY20H080001.

## Conflict of Interest

The authors declare that the research was conducted in the absence of any commercial or financial relationships that could be construed as a potential conflict of interest.

## Publisher’s Note

All claims expressed in this article are solely those of the authors and do not necessarily represent those of their affiliated organizations, or those of the publisher, the editors and the reviewers. Any product that may be evaluated in this article, or claim that may be made by its manufacturer, is not guaranteed or endorsed by the publisher.
